# Management of Lipedema Beyond Liposuction: A Case Study

**DOI:** 10.1093/asjof/ojad088

**Published:** 2023-09-22

**Authors:** Victoria N Bouillon, Chandler S Hinson, MengJie Hu, Ronald M Brooks

## Abstract

Lipedema is a pathologic accumulation of adipose tissue in the subcutaneous layer of the extremities. This connective tissue disorder, which predominately affects females, is often misdiagnosed despite an incidence of ∼11%. Misdiagnosis often leads to delays in appropriate treatment, further increasing the morbidity of the condition. The authors report their facilities' experience in treating a patient with lipedema, requiring multiple surgical interventions involving liposuction and skin debulking to achieve desired aesthetic outcomes. The patient presented to the plastic surgery clinic with severe lipedema of the bilateral lower extremities. She previously underwent a panniculectomy and bilateral lower extremity liposuction without achieving the desired aesthetic results. Prior conservative management and liposuction alone were both unsuccessful treatment options and she required debulking procedures, along with further liposuction, as definitive management. The patient underwent 2 procedures at the clinic, both consisting of liposuction and panniculectomy of the lower extremities and buttocks. The procedures were conducted 1 year apart but were able to achieve the patient's desired aesthetics goals. Management of lipedema can be challenging, but not impossible. This case report shows that local excision is a viable option for treatment if minimally invasive options yield limited results.

**Level of Evidence: 5:**

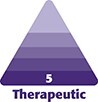

Lipedema is a condition characterized by the abnormal accumulation of excess fibrotic adipose tissue and extracellular fluid in the subcutaneous layer of the limbs, hips, and buttocks. It presents as a symmetric enlargement of limbs with the lower extremities most often affected, resulting in a disproportionate limb-to-torso ratio. It can be accompanied by pain, bruising, impaired mobility, and hypersensitivity to touch, often leading to functional limitations and reduced quality of life.^1^

Although the underlying cause of lipedema remains unclear, genetic and hormonal factors have been implicated. Lipedema has been reported to have a familial component and may be associated with mutations in genes involved in fat metabolism, lymphatic function, and inflammation.^2^ Hormonal imbalances, such as estrogen excess and insulin resistance, may also play a role in the development of this condition.^3,4^ Other hypothesis include primary microvascular dysfunction in both the lymphatic and blood capillaries, causing a hypoxic stimulus that drives excessive expansion of adipose tissue, leading to endothelial dysfunction and increasing angiogenesis.^4,5^ Increased angiogenesis with capillary permeability can lead to the shifting of proteins into the extracellular compartment, leading to fluid expansion in the interstitial space. If this shift is greater than the lymphatic systems ability to clear interstitial fluid, the lymphatic vessels remain enlarged and cause upstream back up leading to enlarging to tissues.^6,7^

Lipedema has an incidence of 6% to 11% with a female predominance.^8^ It is often misdiagnosed as obesity, lymphedema, or cellulitis, leading to delayed diagnosis and inappropriate treatment. The diagnosis is generally made on clinical grounds after exclusion of more common. Diagnostic evaluation consists of a thorough history taking, inspection, and palpation with particular attention to the following manifestations:

- Negative Stemmer sign
- Bilateral, symmetrical, disproportionate fatty tissue hypertrophy on the limbs
- Sparing of hands and feet
- Hematomas or tendency to form hematomas
- Telangiectasias and visible vascular markings around fat deposits
- Pain on pressure and touch

Imaging studies, such as lymphoscintigraphy or MRI, may also be used to support the diagnosis and rule out other conditions.^9^ However, imaging is not necessary for establishing or routinely evaluating lipedema.^10,11^

The management of lipedema is challenging, as there is no definitive cure, and treatment options are largely aimed at improving symptoms and preventing complications. Conservative measures such as compression therapy, manual lymphatic drainage, and exercise may help reduce edema and improve mobility.^12^ However, these approaches are insufficient for addressing the underlying fat accumulation, and surgical intervention is often necessary. Tumescent liposuction has been shown to be effective in reducing the volume of lipedema fat and improving symptoms in both the short and long terms.^13-15^

In recent years, there has been growing interest in the pathophysiology and management of lipedema, with ongoing research aimed at improving our understanding of the disease and developing more effective treatment approaches. Advances in imaging technology, such as lymphoscintigraphy and ultrasound, have helped to improve the accuracy of diagnosis and guide treatment decisions.^16,17^ Furthermore, studies on the use of novel therapies, such as low-level laser therapy, are being conducted to explore their potential for managing lipedema.^18^

In this case report, we provide our facility’s experience in managing a patient with severe lipedema. Written consent was provided by the patient, and the patient agreed to use and analyze their data for this case report. The patient underwent panniculectomy and lymph sparing liposuction, where a total of 18 L of fat was removed from her legs bilaterally. Our patient had both successful cosmetic and functional outcomes, expressing they were able to return to their normal life prior to lipedema.

## CASE PRESENTATION

A 68-year-old female, with a medical history significant for obesity with a BMI of 39.8, presented for evaluation of excess skin to the bilateral lower extremities. The patient had no other chronic comorbidities and was not taking any medications. The patient had undergone a panniculectomy and bilateral lower extremity liposuction 2 years prior to presentation at our clinic. Despite liposuction, the patient continued to experience symptoms secondary to redundant tissue, including multiple skin infections and lower extremity neuropathic pain. Based on this history, medical records, and a thorough physical examination, we had a high clinical suspicion that the patient had lipedema or lymphedema.

**Figure 1.**
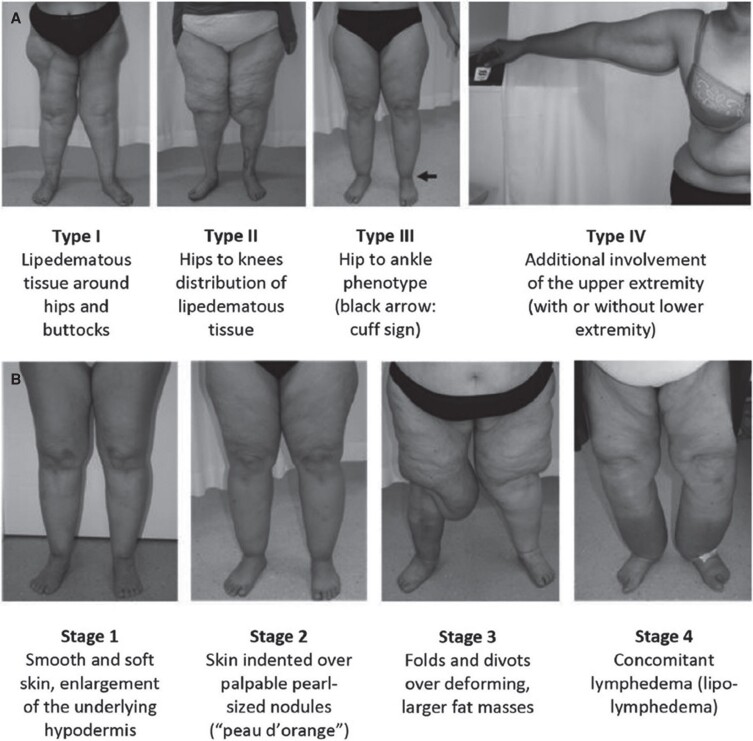
(A) Types and (B) stages of lipedema. Published with permission from Buso et al^19^ under the terms of the Creative Commons Attribution-NonCommercial License (https://creativecommons.org/licenses/by-nc/4.0/).

**Figure 2.**
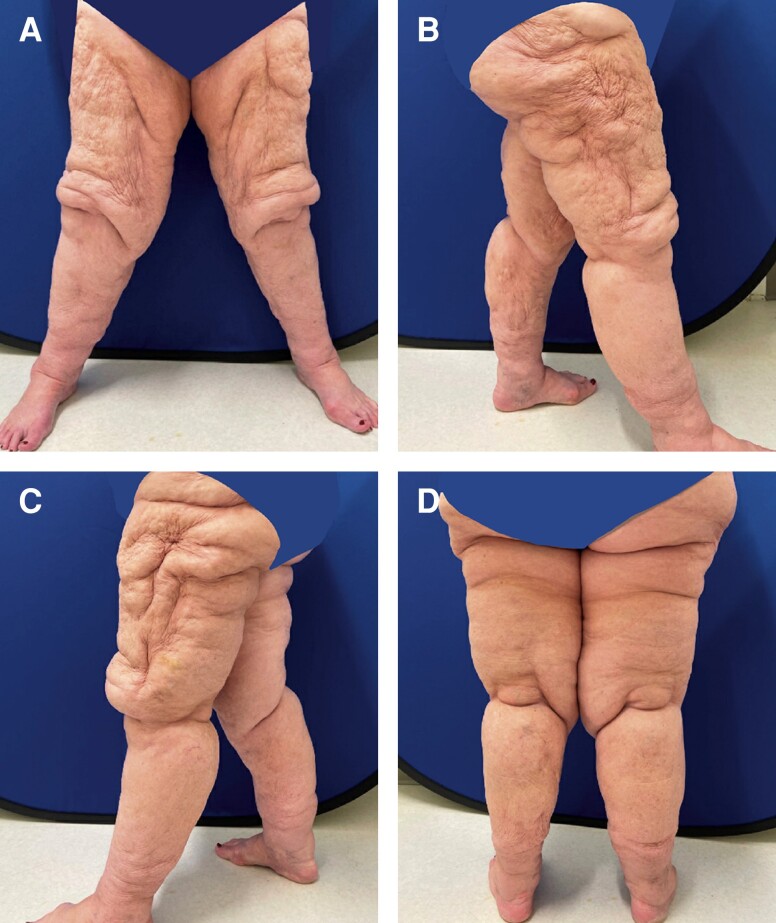
A 68-year-old female patient reported to the plastic surgery clinic with a chief complaint of excessive skin over her bilateral lower extremities. The patient had liposuction on the anterior lower extremities a year prior but stated that she did not achieve the desired aesthetic results. She is shown in (A) frontal view, (B) right lateral view, (C) left lateral view, and (D) posterior view.

The excess skin and subcutaneous tissue, particularly at the groin crease and around the knees, resulted in severely limited mobility, as well as chafing and frequent fungal infections of the inguinal region. On initial examination, she had Type 3, Stage 3 lipedema. Figure 1, adapted from a previous study on lipedema, highlights the stages and types of lipedema, whereas Figure 2 shows the patient at the initial presentation at the clinic.^19^

Preoperative evaluation and marking were performed. Markings were placed with the patient in the standing and supine position. The areas of greatest concern included the length of the medial thighs and the area around the knees. Initial marks were placed along the groin crease following a standard spiral lift pattern. However, the patient had scars from previous surgery in this area; therefore, modifications to our planned incisions were made to optimize both tissue excise and wound healing potential. Ultimately, the patient wished to pursue the most aggressive tissue excision possible. The primary objective in this case was to restore functionality and mobility; cosmesis was not a primary goal. Therefore, tentative circumferential marks around the knees were placed. Furthermore, we anticipated an excess of skin after liposuction that would not be adequately addressed with a spiral lift; therefore, medical vertical incisions were marked. We elected for medial vertical incisions to hide some of the patient's scars.

**Figure 3.**
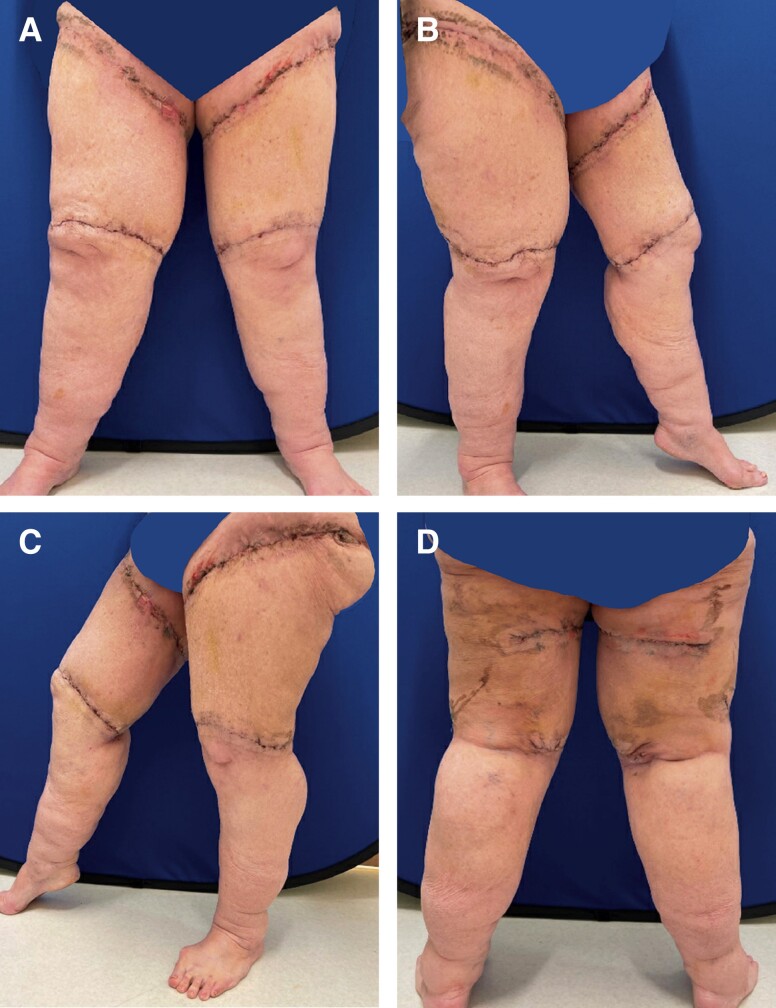
Patient on postoperative Day 14 reporting to clinic with aesthetic satisfaction after procedure for excision of excess skin and soft tissue and tumescent liposuction. 800 cc of tumescent fluid was instilled into each leg, and 1700 cc was extracted from each leg with liposctuion. Skin removal was conducted in the posterolateral buttock area, proximal thighs, and lower knees. She is shown at (A) frontal view, (B) right lateral view, (C) left lateral view, and (D) posterior view.

We began with tumescent liposuction of the bilateral thighs. Lymph sparing techniques were employed as we instilled a greater amount of tumescent fluid than we would have for standard liposuction of the lower extremities. Approximately 800 cc of tumescent fluid was instilled per side. Tumescent was instilled into the posterior thighs, hips, and knees bilaterally. Power-assisted liposuction was used to remove ∼1700 cc from each leg. After liposuction, areas of planned excision about the hip, buttock, proximal thighs, lower knees, and lower legs were pulled taut, and tailor tacking was performed for maximum tissue removal. The incisions along the groin crease and along the upper buttock were created first, and tissue was excised for a thigh lift. To sufficiently address the medial thigh fullness, a vertical incision and tissue excision were performed. An incision around the knees was then created to excise the bulky tissue just proximal to the knee and a medial vertical incision along the calf was made to excise tissue from the lower limbs. Skin closure was conducted without the placement of any drains. Postoperatively, the patient was instructed to wear compressive garments during all waking hours and to elevate the extremities to help with swelling and lymphatic drainage when nonambulatory. Although not a formal recommendation, the patient had a personal lymphatic pump which she began using 3 weeks postoperatively. The patient also underwent regular lymphatic massage. Postoperative cosmetic outcome was excellent and to patient's satisfaction. The patient subsequently developed a minor wound dehiscence secondary to a lymphocele that was successfully managed with local wound care. Figure 3 shows the patient on postoperative Day 14.

Ultimately, the patient had noticeable asymmetry with her right upper leg diameter being slightly larger than the left. Twelve months postoperative, the patient returned to clinic with desire for further debulking to maximize symmetry between lower extremities. Additionally, her lower legs were disproportionately large after the debulking which caused functional problems with mobility and difficulty with finding well-fitting clothing.

**Figure 4.**
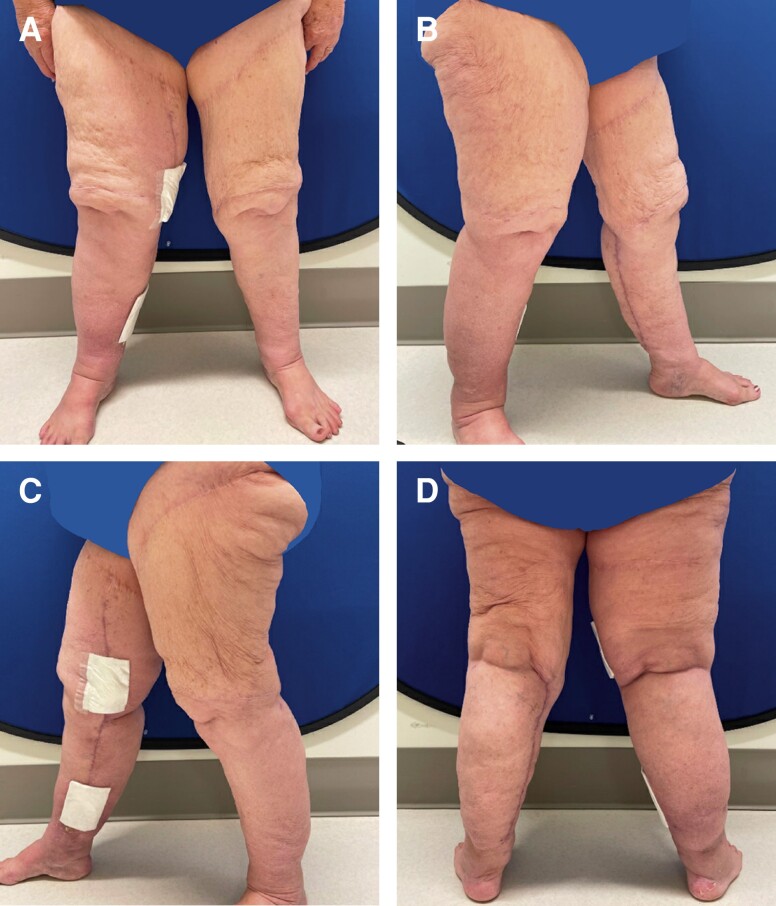
Patient on postoperative Day 12 after the second procedure for further debulking and liposuction. 1400 cc of tumescent fluid was instilled into both legs, and 2000 cc was extracted from the right and 350 cc was extracted from the lower left leg. The patient was pleased with the final aesthetic results. She is shown in (A) frontal view, (B) right lateral view, (C) left lateral view, and (D) posterior view.

Approximately 15 months after the initial bilateral lower extremity liposuction and thigh lift, she underwent further liposuction and the excision of excess skin and soft tissue of the bilateral lower extremities. Approximately 1400 cc of tumescent fluid was instilled into both legs, and 2000 cc was extracted from the right leg with liposuction. Approximately 350 cc was extracted from the lower left leg. Liposuction and skin excision were performed similarly to the first procedure described above; however, closure included the placement of 19 JP French drains bilaterally. These drains were left in place and removed on postoperative Day 10. Resultant symmetry was improved. Surgical pathology showed benign skin and subcutaneous tissue. Figure 4 shows patient 12 days postoperative.

**Figure 5.**
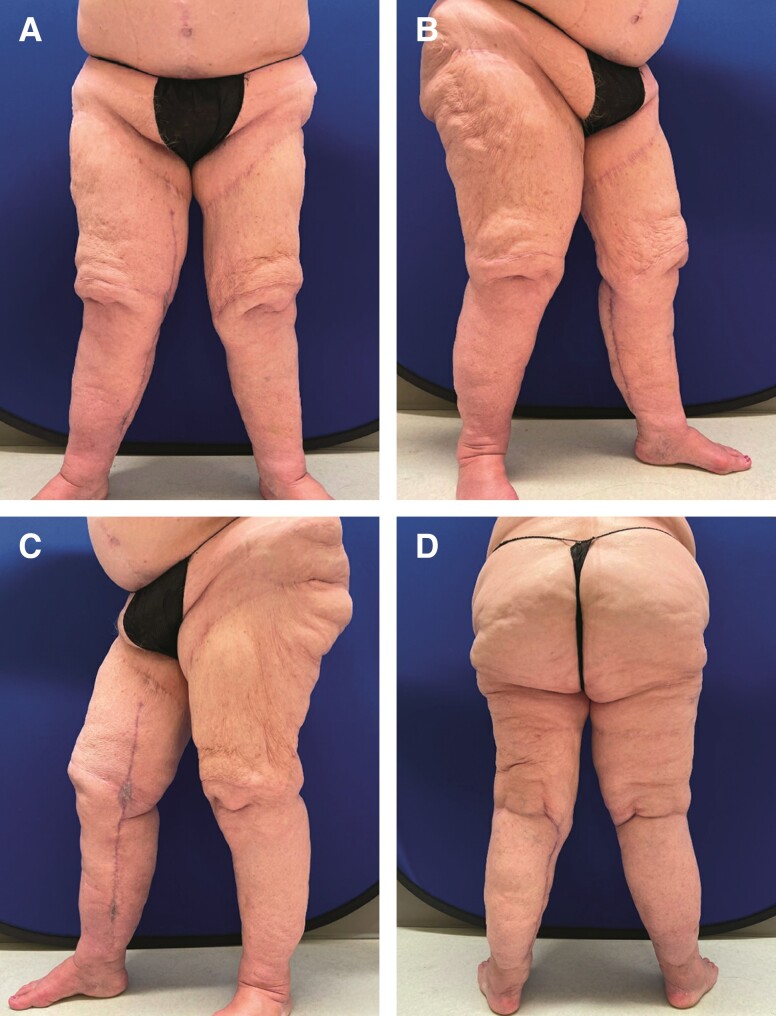
Patient postoperative 3 months after the final debulking and liposuction procedure. The patient was satisfied with the final results, with the patient stating she had both a functional and cosmetic improvement from the initial presentation 2 years prior. She is shown in (A) frontal view, (B) right lateral view, (C) left lateral view, and (D) posterior view.

The patient returned to the emergency department on postoperative Day 15 with cellulitis and pain from the medial incisions. A conservative course of outpatient antibiotics was trialed with minimal improvement, and she was ultimately admitted for intravenous antibiotics. Cross-sectional imaging revealed multiple fluid-filled collections, and therefore, a formal incision and drainage procedure were performed. She was noted to have a hematoma without purulence or gross signs of infection within the wound. After drainage and a course of intravenous antibiotics, the patient progressed well and was discharged with a short course of antibiotics and local wound-care instructions. The patient's wounds progressed well and had no further complications. The patient’s last follow-up, which was postoperative 3 months, stated satisfaction with cosmetic and functional results. Photographs at postoperative 3 months are shown in Figure 5.

## DISCUSSION

Lipedema is a loose connective tissue disease that predominantly affects females. The symmetric enlargement of the extremities is due to disproportionate deposits of adipose tissue, causing a decreased ability to ambulate the lower extremities. Additionally, lipedema increases the risk of bacterial and fungal skin infections.

The diagnosis of lipedema can be difficult and is often a diagnosis of exclusion. It is most commonly mistaken for simple obesity.^20^ Other pathologies that lie on the differential include lymphedema, lipolymphedema, lipohypertrophy, phlebedema, venolipolyphedema, and gynecoid obesity.^20,21^ Moreover, the pathophysiology of these conditions are interconnected, and multiple diagnoses can coexist, further complicating diagnosis. Multiple imaging modalities including ultrasonography, lymphoscintigraphy, and MRI have been employed to facilitate diagnosis of lipedema; however, diagnostic imaging is lacking, and ultimately diagnosis is based on clinical examination.^22^

Treatment focuses on symptom reduction, relief of functional limitations, and prevention of disease progression. Therapy targeting disease etiology does not yet exist.^22^ Conservative management, which includes exercise, lymphatic massage, and compressive garments, is trialed first. These measures address factors thought to impact the progression of lipedema—obesity and anomalous lymphovascular drainage.^20,22^ The efficacy of these therapies is limited, as they do very little to address adipose tissue but may be beneficial in pain and edema reduction. The next step in management is tumescent liposuction. Long-term studies have found it to be quite successful in symptom relief and slowing disease progression.^22^ Compared with dry liposuction, the use tumescence has been shown to preserve lymphatics. Dependent of the extent of lipedema, multiple treatments are usually indicated. Ultimately, if liposuction alone is unsuccessful, debulking procedures can be performed.^21,22^ One pitfall of debulking is the risk of damage to the lymphatics which can thus further exacerbate edema. Wound healing is always a concern after debulking given the pathophysiology of the disorder, and therefore, selective excision of symptomatic regions is favored. This was a special case in that this patient had a significant amount of excess tissue afflicting the area around her knees that was functionally limiting as well as cosmetically displeasing. While not conventional, in order to adequately treat her symptoms, circumferential knee incisions were necessary. She did not experience wound complications or adverse sequelae of the resultant scars. In general, we do not have a large patient population who are afflicted by excess tissue in a similar distribution as the patient discussed in this paper. Furthermore, most of our patients are quite particular about scarring and would not want circumferential knee scars. This patient did not care about scarring. She desired an aggressive excision to relieve her symptoms.

Our patient failed conservative management and experienced progression of disease despite treatment with liposuction. Debulking of excess skin and soft tissue was effective in treating her functional symptoms as well as pain and edema. Our patient experienced wound healing complications, requiring drainage and both intravenous and outpatient antibiotics to control the infection. Past the complications, the patient has not had further reaccumulation of adipose tissue in the affected areas. The patient continues to remain satisfied with her aesthetic results from the procedures. These results are encouraging, but the team recognizes that they are incomplete as they are currently 3 months postoperative. The results can change drastically within the upcoming months. While this is a limitation of this case report, it is important to recognize both the aesthetic and functional satisfaction of the patient.

## CONCLUSIONS

Lipedema is a lifelong disorder of loose connective tissues. Aberrant fat deposits can reaccumulate at sites of previous excision, or more commonly, new areas that were previously asymptomatic may become problematic or more apparent after surgery. Diagnosis is complex, yet critical for adequate treatment. Management can be challenging, and multiple procedures may be warranted. Ultimately, therapy should be individualized and should start with conservative measures, increasing the rigor based on patients' response to therapy. However, more aggressive treatment with liposuction and lipectomy should not be postponed if conservative treatment fails. With our patient, aggressive treatment allowed the patient to be satisfied with her final aesthetic result.
